# Evidence That ADAM17 Mediates the Protective Action of CGRP against Angiotensin II-Induced Inflammation in Vascular Smooth Muscle Cells

**DOI:** 10.1155/2018/2109352

**Published:** 2018-06-12

**Authors:** Si-yu Zeng, Li Yang, Chen-liang Hong, Hui-qin Lu, Qiu-jiang Yan, Yan Chen, Xu-ping Qin

**Affiliations:** ^1^Institution of Drug Clinical Trial, Guangdong Second Provincial General Hospital, Guangzhou 510317, China; ^2^Laboratory of Vascular Biology, Institute of Pharmacy and Pharmacology, University of South China, Hengyang, 421001 Hunan, China; ^3^Department of Cardiac & Thoracic Surgery, The Third Affiliated Hospital of Guangzhou Medical University, Guangzhou 510000, China

## Abstract

Calcitonin gene-related peptide (CGRP) has a potent protective action on the cardiovascular system; however, little is known about the role of CGRP in angiotensin II- (Ang II-) induced inflammation of vascular smooth muscle cells (VSMCs). This study is aimed at determining the anti-inflammatory effect of CGRP in Ang II-treated VSMCs and whether a disintegrin and metalloproteinase 17 (ADAM17) modulates this protective action. Small interference RNA (siRNA) and inhibitors of CGRP, epidermal growth factor receptor (EGFR), and extracellular signal-regulated kinase 1/2 (ERK1/2) were adopted to investigate their effect on Ang II-induced inflammation in VSMCs. Here, we found that CGRP could inhibit inflammation and decrease ADAM17 expression and activation of EGFR and ERK1/2 in VSMCs stimulated with Ang II. Results of siRNA demonstrated that ADAM17 siRNA attenuated Ang II-induced inflammation and up-regulation of activities of EGFR and ERK1/2 in VSMCs. Furthermore, the EGFR-ERK1/2 pathway promoted Ang II-induced VSMC inflammation. In summary, these findings identify the anti-inflammatory effect of CGRP in VSMCs stimulated by Ang II and suggest that ADAM17 is involved in the protective effect of CGRP against Ang II-induced inflammation via the EGFR-ERK1/2 pathway in VSMCs.

## 1. Introduction

Chronic vascular inflammation contributes to the initiation, development, and progression of a series of cardiovascular diseases including hypertension and atherosclerosis. Proinflammatory cytokines such as tumor necrosis factor *α* (TNF-*α*), interleukin 6 (IL-6), and c-reactive protein (CRP) have been recognized as the markers of inflammation [1]. In addition to leukocytes, vascular smooth muscle cells (VSMCs) could be another crucial source of proinflammatory cytokines in the vessel wall [2, 3]. *In vivo* and *in vitro* studies show that angiotensin II (Ang II) induces the expression of proinflammatory cytokines in the vasculature such as IL-6, vascular cell adhesion molecule-1 (VCAM-1), and monocyte chemoattractant protein-1 (MCP-1) [2–4]. Thus, Ang II-mediated VSMC inflammation plays a key role in the development of cardiovascular diseases; however, its mechanisms remain to be incompletely elucidated.

Calcitonin gene-related peptide (CGRP), a 37-amino acid neuropeptide, is the most potent vasodilated vasodilator secreted by the sensory nerve terminal. Previous studies have demonstrated that CGRP could protect against cardiovascular diseases such as hypertension and heart failure [5–8]. Recently, CGRP is also considered as a critical proinflammatory neuropeptide in the pathophysiology of migraine [9]. Furthermore, macrophage infiltration and TNF-*α* production were elevated within the laser-induced lesions of choroidal neovascularization in CGRP (−/−) mice [10]. These results suggest the possibility that the anti-inflammatory effect of CGRP might play an important role in the development of cardiovascular diseases; nevertheless, little is known about the relation between CGRP and inflammation in VSMCs.

A disintegrin and metalloproteinase 17 (ADAM17), also known as tumor necrosis factor-*α*-converting enzyme (TACE), promotes cardiovascular remodeling that plays a crucial role in cardiovascular diseases [11–15]. This enzyme has essential functions in cell-cell interactions, in signaling, and in proteolysis of cytokines, cytokine receptors, and other targets. Activated ADAM17 could induce the release of TNF-*α* and soluble IL-6 receptor (IL-6R) that forms a complex by binding with IL-6 [1, 15, 16]. However, the role of ADAM17 in VSMC inflammation has not been determined, nor has it been elucidated whether ADAM17 regulates the protective effect of CGRP against Ang II-induced VSMC inflammation.

In this study, we establish that CGRP attenuates inflammation by decreasing the expression of IL-1*β*, IL-6, and TNF-*ɑ* in VSMCs treated with Ang II, and ADAM17 mediates this anti-inflammatory effect of CGRP through the EGFR-ERK1/2 pathway.

## 2. Methods and Materials

### 2.1. Cell Culture

Cell lines of VSMCs, derived from thoracic artery in rats, were originated from the ATCC cell bank (Manassas, VA, America). These cells were placed and cultured in normal condition (37°C, 5% carbon dioxide) with Dulbecco modified Eagle's medium (DMEM, Thermo Fisher, America) containing 10% fetal bovine serum (FBS). When VSMCs reach at about 80% confluence, these cells were needed to incubate for 24 hours in DMEM with 0.1% FBS before stimulation.

### 2.2. RNA Interference

Small interference RNA (siRNA) sequences against rat ADAM17 were synthesized by Shanghai GenePharma. The ADAM17 antisense sequence is 5′-ACUUCACACUGUA CUCGCUTT-3′, while the scrambled sequence was used as a negative control (NC). Small interference RNA (100 nmol/l) was transiently transfected into the cells via adding it to 5 *μ*l Lipofectamine 2000 (Invitrogen, America) per 20 mm dish according to the manufacturer's instruction.

### 2.3. Western Blotting

Protocols of Western blotting were referred by our previous reference [17]. Antibodies were described as follows: ADAM17 antibody (Abcam, Britain), phosphor-EGFR (p-EGFR) (Abcam, Britain), EGFR (Abcam, America), phosphor-ERK1/2 (p-ERK1/2) (Abcam, Britain), ERK1/2 (Abcam, America), and Goat Anti-Rabbit IgG HRP (Affinity, America). Relative levels of immunoreactive proteins were detected by chemiluminescence and quantified with ImageJ software.

### 2.4. Enzyme-Linked Immunosorbent Assay (ELISA)

After cells were stimulated with Ang II for 24 hours, the conditioned medium was collected to detect the mature IL-1*β*, IL-6, and TNF-*α* released from VSMCs. ELISA kits (Cusabio, China) was used to determine the protein levels of IL-1*β*, IL-6, and TNF-*α* in culture media according to the manufacturer's instructions, briefly described as follows: First, the standard was reconstituted with 1.0 ml of sample diluent to produce a stock solution, used to produce a 2-fold dilution series, and the sample diluent served as the zero standard (0 pg/ml). After treating with the corresponding biotin antibody, HRP Avidin, and TMB substrate, these 2-fold dilution series were adopted to determine the optical density of each well within 5 minutes under 450 nm using an automatic enzyme-linked immunoadsorbent assay system. Second, the concentration of 2-fold dilution series were plotted against the corresponding optical density resulting in a standard curve used to determine the protein level of IL-1*β*, or IL-6, or TNF-*α* in the conditioned medium. Third, the same procedures were adopted to determine the optical density in the conditioned medium, and then the concentration of IL-1*β*, or IL-6, or TNF-*α* was calculated.

### 2.5. Real-Time Quantitative PCR

Total cellular RNA was prepared using TRIzol purchased from Takara according to the instruction. The reverse transcription system (Takara, Japan) was used to synthesize the first-strand complementary DNA. Real-time PCR used primers for ADAM17, IL-1*β*, IL-6, and TNF-*α* in accordance with the manufacturer's instruction (Takara, Japan), and the gene specific for GAPDH was chosen as an inner control. Primers for ADAM17 are 5′-GTGAGCAGTTTCTCGAACGC-3′ (forward primer) and 5′-AGCTTCTC AAGTCGCAGGTG-3′ (reverse primer); primer for IL-1*β* are 5-TCCTCTGTGACTCG TGGGAT-3 (forward primer) and 5′-TCAGACAGCACGAGGCATTT-3′ (reverse primer); primers for IL-6 are 5′-TCCTACCCCAACTTCCAATGCTC-3′ (forward primer) and 5′-TTG GATGGTCTTGGTCCTTA GCC-3′ (reverse primer); primers for TNF-*α* are 5′-TGGCGT GTTCATCCGTTCTC-3′ (forward primer) and 5′-CCCAGAGCCACAATTCCCTT-3′ (reverse primer); and primers for GADPH are 5′-ATCAAGAAGGTGGTGAAGCA-3′ (forward primer) and 5′-AAGGTGGAAGAATGG GAGTTG-3′ (reverse primer).

### 2.6. Statistical Analysis

Quantitative variables are presented as mean ± standard deviation (SD). Statistical significance between 2 means was performed by unpaired *t*-test, whereas those among more than 2 means with two independent variables were by two-way ANOVA with Bonferroni posttest. *P* values of <0.05 were considered to have statistical significance.

## 3. Results

### 3.1. CGRP Protected against Ang II-Induced Inflammation in VSMCs

**Figure 1:**
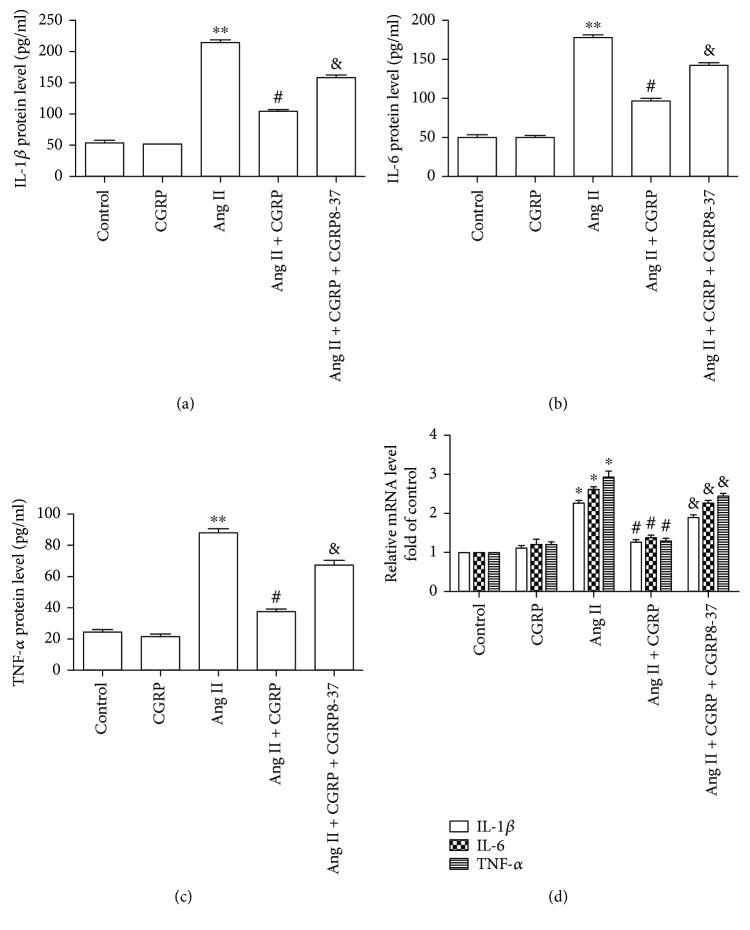
CGRP dampened Ang II-induced inflammation in vascular smooth muscle cells. After pretreating with 10 nmol/l CGRP or/and 50 nmol/l CGRP8-37 for 30 minutes, vascular smooth muscle cells were stimulated with 100 nmol/l Ang II for 24 hours, then the medium was collected to assay for the concentration of IL-1*β*, IL-6, and TNF-*α* by ELISA, while the cells were adopted to measure the mRNA levels of IL-1*β*, IL-6, and TNF-*α*. (a–c) the protein levels of IL-1*β* (a), IL-6 (b), and TNF-*α* (c), *n* = 3–5 independent experiments. (d) The mRNA levels of IL-1*β*, IL-6, and TNF-*α*, *n* = 3 − 4 independent experiments. Ang II represents angiotensin II. ^∗^ *P* < 0.05 versus control, ^∗∗^ *P* < 0.01 versus control, ^#^ *P* < 0.05 versus Ang II, and ^&^ *P* < 0.05 versus Ang II + CGRP.

As shown in Figure 1, Ang II induced the release of IL-1*β*, IL-6, and TNF-*α* from VSMCs and the increase in mRNA levels of IL-1*β*, IL-6, and TNF-*α* compared with the control group. Pretreatment with CGRP inhibited Ang II-induced upregulation of protein release and mRNA levels of IL-1*β*, IL-6, and TNF-*α*, whereas these effects were canceled by CGRP antagonist CGRP 8-37. These data suggest that CGRP could attenuate Ang II-induced inflammation in VSMCs.

### 3.2. ADAM17 Mediated the Anti-Inflammatory Effect of CGRP

**Figure 2:**
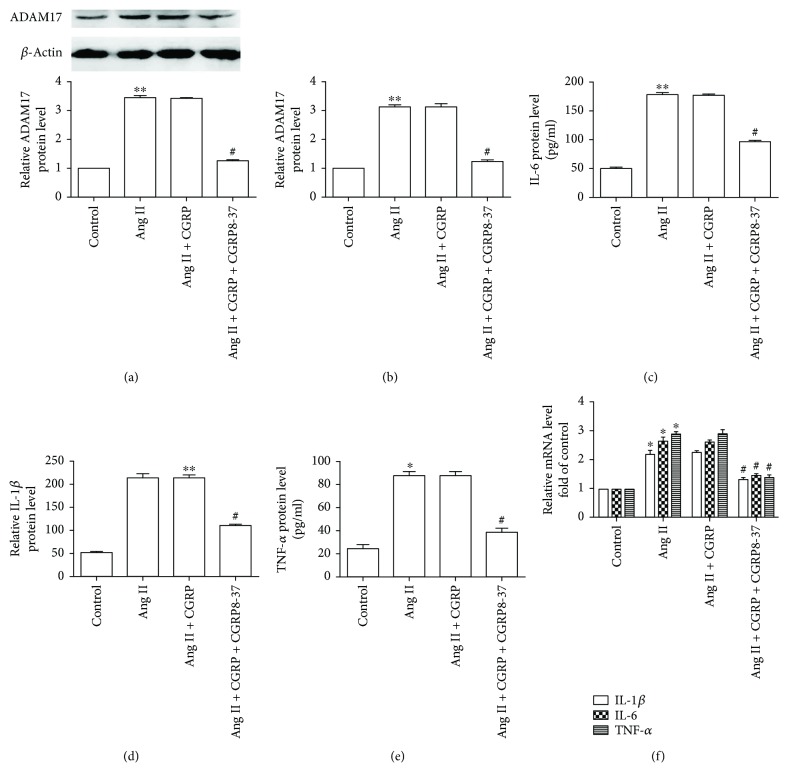
ADAM17 siRNA attenuated Ang II-induced inflammation in vascular smooth muscle cells. After being transfected with 100 nmol/l ADAM17 siRNA for 30 hours, vascular smooth muscle cells were then stimulated with 100 nmol/l Ang II for 24 hours. (a) ADAM17 protein level, *n* = 4 independent experiments. (b) ADAM 17 mRNA level, *n* = 3 independent experiments. (c–e) At the end of the experiment, the medium was collected to detect the concentration of IL-1*β*, IL-6, and TNF-*α* by ELISA, *n* = 3–5 independent experiments. (c) IL-1*β* protein level. (d) IL-6 protein level. (e) TNF-*α* protein level. (f) The mRNA levels of IL-1*β*, IL-6, and TNF-*α*, *n* = 3 − 4 independent experiments. Ang II: angiotensin II; NC: negative control. ^∗^ *P* < 0.05 versus control, ^∗∗^ *P* < 0.01 versus control, and ^#^ *P* < 0.05 versus Ang II.

**Figure 3:**
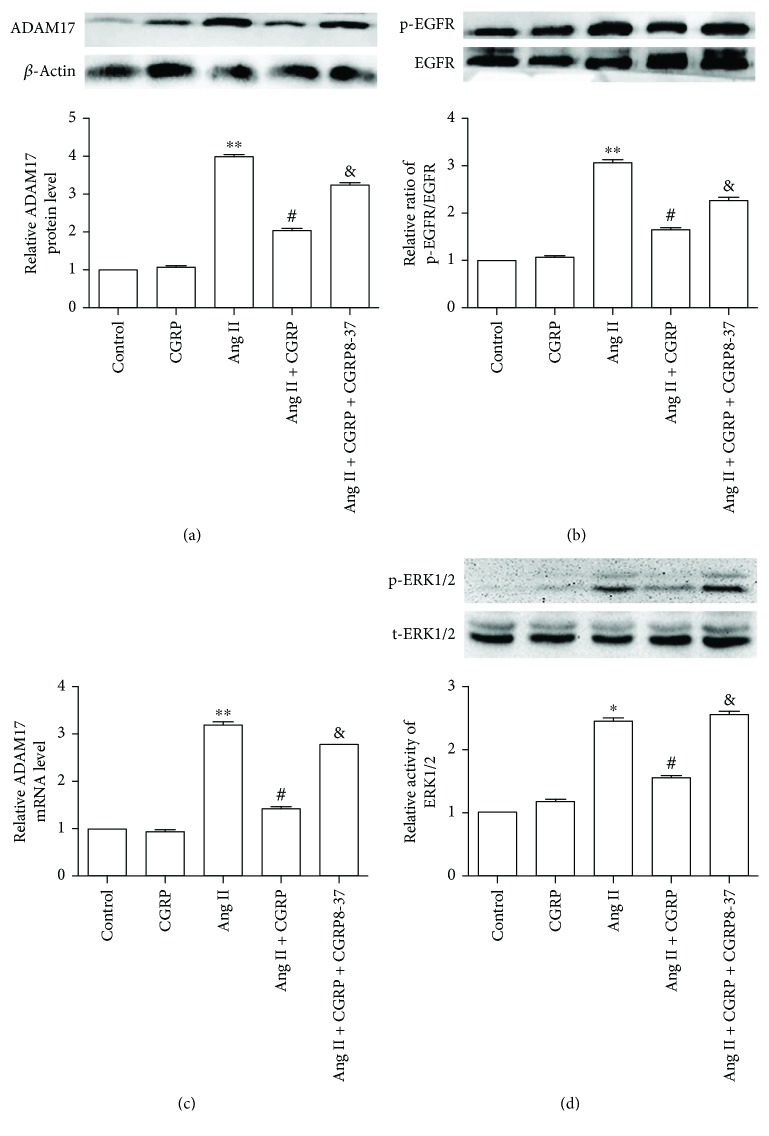
CGRP reduced ADAM17 expression and activation of EGFR and ERK1/2 in vascular smooth muscle cells treated with Ang II. After being pretreated with 10 nmol/l CGRP or/and 10 nmol/l CGRP 8–37 for 30 minutes, vascular smooth muscle cells were treated with 100 nmol/l Ang II for 24 hours. (a) ADAM17 protein level, *n* = 4 independent experiments. (b) ADAM17 mRNA level, *n* = 3 independent experiments. (c) EGFR activity, *n* = 4 independent experiments. (d) ERK1/2 activity, *n* = 3 independent experiments. Ang II represents angiotensin II. ^∗^ *P* < 0.05 versus control, ^∗∗^ *P* < 0.01 versus control, ^#^ *P* < 0.05 versus Ang II, and ^&^ *P* < 0.05 versus Ang II + CGRP.

Technology of RNA interference was adopted to observe the effect of ADAM17 on Ang II-induced inflammation. The representative photos of VSMCs transfected with siRNA marked with green fluorescent protein are shown in Figure S1. As displayed in Figures 2(a) and 2(b), ADAM17 siRNA significantly reduced ADAM17 mRNA and protein levels in VSMCs, demonstrating the validity of the siRNA. Compared with the Ang II group, ADAM17 siRNA remarkably diminished the protein release and mRNA expression of IL-1*β*, IL-6, and TNF-*α* in VSMCs stimulated by Ang II (Figures 2(c)–2(f)). These results showed that ADAM17 promoted Ang II-induced VSMC inflammation. Moreover, CGRP could significantly decrease the ADAM17 protein and mRNA levels in VSMCs treated with Ang II for 24 hours (Figures 3(a) and 3(b)). Overall, ADAM17 modulated the protective effect of CGRP on Ang II-induced inflammation in VSMCs.

### 3.3. EGFR-ERK1/2 Signaling Pathway Modulated the Protective Action of CGRP against Ang II-Induced Inflammation in VSMCs

**Figure 4:**
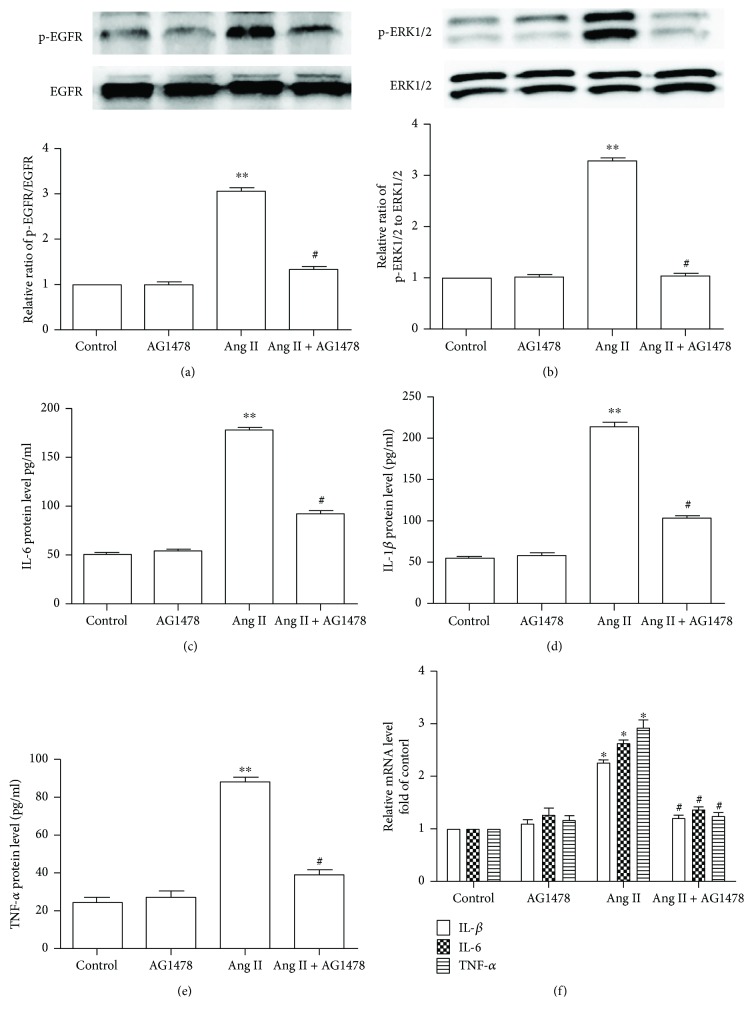
EGFR activation is required in Ang II-induced inflammation in vascular smooth muscle cells. After being pretreated with selective EGFR inhibitor AG1478 (5 *μ*mol/l) for 30 minutes, vascular smooth muscle cells were then stimulated with 100 nmol/l Ang II for 24 hours. (a) EGFR activity, *n* = 4 independent experiments. (b) ERK1/2 activity, *n* = 3 independent experiments. (c–e) At the end of the experiment, the medium was collected to detect the concentration of IL-1*β*, IL-6, and TNF-*α* by ELISA, *n* = 3–5 independent experiments. (c) IL-1*β* protein level. (d) IL-6 protein level. (e) TNF-*α* protein level. (f) The mRNA levels of IL-1*β*, IL-6, and TNF-*α*, *n* = 3 − 4 independent experiments. Ang II represents angiotensin II. ^∗^ *P* < 0.05 versus control, ^∗∗^ *P* < 0.01 versus control, and ^#^ *P* < 0.05 versus Ang II.

**Figure 5:**
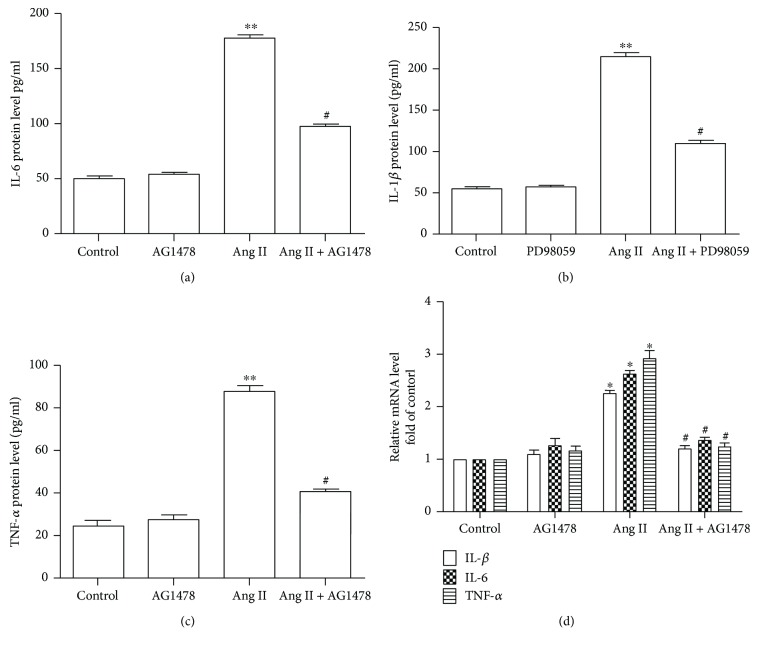
ERK1/2 activation mediates Ang II-induced inflammation in vascular smooth muscle cells. After being pretreated with ERK1/2 inhibitor PD98059 (10 *μ*mol/l) for 30 minutes, vascular smooth muscle cells were stimulated with 100 nmol/l Ang II for 24 hours. (a–c) At the end of the experiment, the medium was collected to detect the concentration of IL-1*β*, IL-6, and TNF-*α* by ELISA. (a–c) the protein levels of IL-1*β* (a), IL-6 (b), and TNF-*α* (c), *n* = 3–5 independent experiments. (d) The mRNA levels of IL-1*β*, IL-6, and TNF-*α*, *n* = 3-4 independent experiments. Ang II represents angiotensin II. ^∗^ *P* < 0.05 versus control, ^∗∗^ *P* < 0.01 versus control, and ^#^ *P* < 0.05 versus Ang II.

Pretreatment with CGRP for 30 minutes markedly decreased the phosphorylation levels of EGFR and ERK1/2 in VSMCs treated with Ang II for 24 hours, whereas CGRP8-37 canceled these effects (Figures 3(c) and 3(d)). Subsequently, EGFR inhibitor AG1478 and ERK1/2 antagonist PD98059 were used to test whether the EGFR-ERK1/2 pathway advanced Ang II-induced inflammation.  Figure 4 shows that Ang II significantly elevated the protein release and mRNA levels of IL-1*β*, IL-6, and TNF-*α* and the ERK1/2 phosphorylation level compared with the control group, whereas pretreating with a selective EGFR inhibitor AG1478 for 30 minutes reversed these effects. Next, the results of ERK1/2 antagonist PD98059 intervention indicated that ERK1/2 activation promoted Ang II-induced elevation of protein release and mRNA levels of IL-1*β*, IL-6, and TNF-*α* in VSMCs (Figure 5). Collectively, CGRP alleviated Ang II-induced inflammation via inhibiting the EGFR-ERK1/2 pathway in VSMCs.

### 3.4. ADAM17 Is Required in the Activation of the EGFR-ERK1/2 Pathway Induced by Ang II

**Figure 6:**
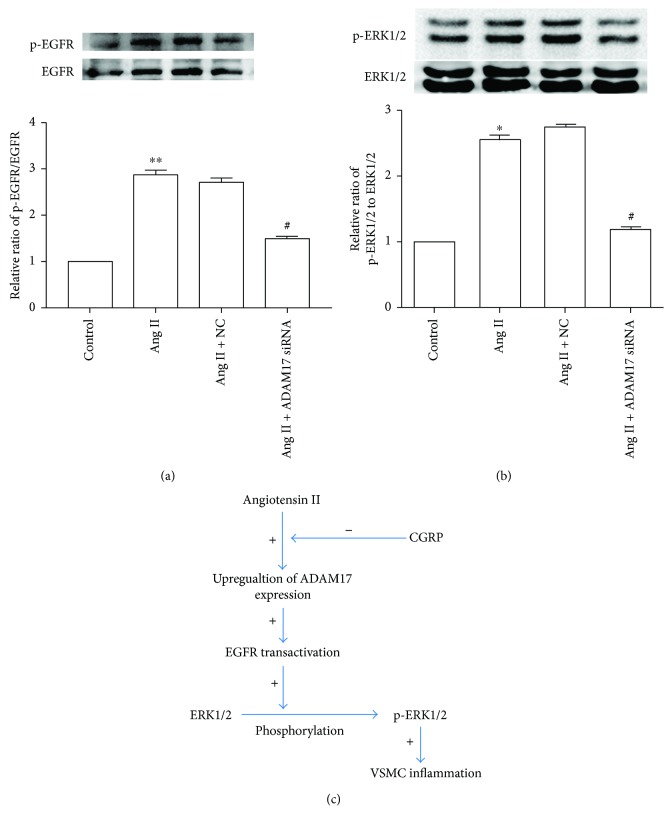
ADAM17 siRNA decreased Ang II-induced activation of EGFR and ERK1/2 in vascular smooth muscle cells. After being transfected with 100 nmol/l ADAM17 siRNA for 30 hours, vascular smooth muscle cells were then stimulated with 100 nmol/l Ang II for 24 hours. (a) EGFR activity, *n* = 4 independent experiments; (b) ERK1/2 activity, *n* = 3 independent experiments; (c) proposed model of ADAM17 in regulating the inhibitory effect of CGRP on Ang II-induced inflammation through the EGFR-ERK1/2 pathway. Ang II: angiotensin II; NC: negative control; +: positive effect; −: negative effect; VSMCs: vascular smooth muscle cells. ^∗^ *P* < 0.05 versus control, ^∗∗^ *P* < 0.01 versus control, and ^#^ *P* < 0.05 versus Ang II.

To determine the relationship of ADAM17 and activation of EGFR and ERK1/2, ADAM17 siRNA was used in the present study. As shown in Figure 6, Ang II-induced EGFR and ERK1/2 activation was attenuated by treatment of ADAM17 siRNA in VSMCs, suggesting that the activation of the EGFR-ERK1/2 pathway induced by Ang II is mediated by ADAM17.

## 4. Discussion

The mechanisms of Ang II-mediated VSMC inflammation includes NF-*κ*B activation and toll-like receptor 4 (TLR4) expression. NF-*κ*B activation plays a central role in Ang II-induced IL-6 expression in VSMCs [18, 19]. In additional to NF-*κ*B, Ang II-induced upregulation of IL-6 expression still requires cAMP response element-binding protein (CREB) and ERK-dependent histone acetylation mediated by p300 and steroid receptor coactivator-1 (SRC-1) [20]. Pretreatment with anti-TLR4 antibody, TLR4 inhibitor, or TLR4 siRNA prior to Ang II stimulation significantly diminished VSMC inflammation [21, 22]. In the present study, results show that Ang II induces inflammation of VSMCs by increasing ADAM17 expression and EGFR activation, presenting a new sight for understanding Ang II-induced VSMC inflammation.

EGFR transactivation plays a key role in Ang II-induced VSMC inflammation associated with cardiovascular diseases. EGFR transactivation has been implied in several cardiovascular conditions, including hypertension, heart failure, and cardiac and vascular hypertrophy [23–26]. It is reported that loss of vascular smooth muscle cell-EGFR increases mRNA levels of proinflammatory cytokines (such as MCP-1 and TNF-*α*) in the aorta, indicating that maintaining a certain activity of EGFR in vasculature is required in physiological conditions [27]. Our results showed that inhibition of EGFR could attenuate inflammation in VSMCs treated by Ang II. These results indicated that activation of EGFR might be a two-edged sword; excessive EGFR activation might produce harmful effects to cardiovasculature. EGFR deletion significantly decreases ERK1/2 phosphorylation level induced by endothelin 1 or *α*1-adrenoceptor or oxidative stress in VSMCs [28], which is inconsistent with our finding that EGFR activation could promote Ang II-induced ERK 1/2 activation. Moreover, our data also showed that inhibition of ERK1/2 inhibited Ang II-induced inflammation in VSMCs. Overall, the EGFR-ERK1/2 pathway plays an important role in Ang II-induced inflammation.

ADAM17 liberates and activates EGFR ligands from their membrane anchor, such as transforming growth factor-*α* (TGF-*α*), amphiregulin, and heparin-binding EGF-like growth factor (HB-EGF). A growing number of studies reported that ADAM17 induces EGFR transactivation in different types of cells such as vascular smooth muscle cell [29], cardiac cell [17], hepatic stellate cell [30], and endothelial cell [31], and the same effect was also observed in our results as well. Further, our findings suggested that ADAM17 siRNA decreased Ang II-induced inflammation and ERK1/2 activation in VSMCs. Therefore, ADAM17 induces Ang II-induced inflammation through the EGFR-ERK1/2 pathway in VSMCs.

CGRP, including *α*-CGRP and *β*-CGRP, has a potent protection for the cardiovascular system. The results of *α*-CGRP knockout and analogue demonstrate the protective action of *α*-CGRP against vascular remodeling in Ang II-induced hypertension [5, 8]. Our previous results showed that endogenous CGRP involves in the depressor effect and regression of vascular remodeling of losartan or perindopril in 2-kidney, 1-clip hypertensive rats [32]. The inhibitory effect of CGRP on VSMC proliferation constitutes the basis of the protective action of CGRP against vascular remodeling [33, 34], and the mechanisms include ERK1/2 activation, P53, and cAMP/protein kinase A (PKA) [33, 35] Our findings showed that CGRP inhibited Ang II-induced inflammation in VSMCs, providing a new insight in understanding the protective action of CGRP against Ang II-induced vascular remodeling. Further research, however, is still needed to elucidate about the mechanisms of anti-inflammatory action of CGRP in VSMCs stimulated with Ang II. In the present study, indirect evidences showed that ADAM17 negatively modulated the protective effect of CGRP against Ang II-induced inflammation through the EGFR-ERK1/2 pathway.

## 5. Conclusions

In summary, we identify the anti-inflammatory effect of CGRP on Ang II-induced VSMC inflammation, and indirect evidences indicate that ADAM17 mediates the protective effect of CGRP against Ang II-induced inflammation through the EGFR-ERK1/2 pathway in VSMCs (Figure 6(c)). This will help us better understand the mechanisms of VSMCs inflammation and how CGRP protects against cardiovascular disease resulting from the overactivation of the rennin-angiotensin system, providing further basis for ADAM17 and CGRP as potential targets against vascular inflammation and cardiovascular diseases. Nevertheless, further research is still needed to demonstrate these arguments in CGRP^−/−^ animals and elucidate how CGRP mediates ADAM17 expression.

## Data Availability

All data used to support the findings of this study are available from the corresponding authors upon request.

## Supplementary Materials

Supplementary Materials Figure S1: representative microphotographs of vascular smooth muscle cells transfected with siRNA after being transfected with 100 nmol/l siRNA marked with green fluorescent protein for 12 hours. The photos of these cells were taken using a fluorescence microscope under 40× amplification.
