# Supplementary material for: Evidence That ADAM17 Mediates the Protective Action of CGRP against Angiotensin II-Induced Inflammation in Vascular Smooth Muscle Cells
Source: Mediators Inflamm. 2018 Jun 12;2018:2109352. doi: 10.1155/2018/2109352 (PMC6038660; doi:10.1155/2018/2109352)
Supplement: Supplementary Materials — Figure S1: representative microphotographs of vascular smooth muscle cells transfected with siRNA after being transfected with 100 nmol/l siRNA marked with green fluorescent protein for 12 hours. The photos of these cells were taken using a fluorescence microscope under 40× amplification. [file 2109352.f1.docx]

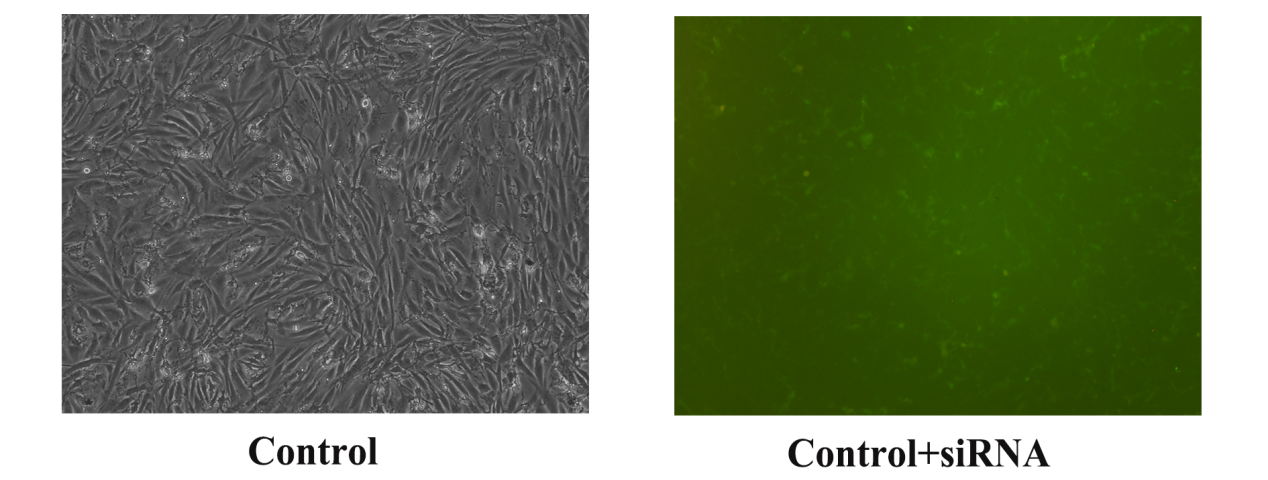


**Figure S1** Representative microphotographs of vascular smooth muscle cells transfec -ted with siRNA. After transfected with 100 nmol/L siRNA marked with green fluore- scent protein for 12 hours, these cells were taken photos using fluorescence microscope under the amplification times ( 40×).
